# An Employee's Industrial Views

**Published:** 1920-05-01

**Authors:** Theodore Harris

**Affiliations:** 180 Uttoxeter Old Road, Derby.


*To the Editor of* THE HOSPITAL.

*To the Editor of* THE HOSPITAL.

Yours faithfully,

GLADYS M. E. LEIGH.

### An Employee's Industrial Views.

*To the Editor of* THE HOSPITAL.

SIR,—I beg to call your attention to an advertisement in your columns of April 24 headed Borough of Bermondsey, inviting applications for the appointment of two midwives. The advertisement states that candidates are to hold the certificate of the Central Midwives Board, be registered midwives, *and will be required to belong to a trade union.* (The italics are my own.) Surely a midwife has every right to belong or not to a trade union as she wishes. It is the affair of the Council that she be well and fully trained, and that she will act to the best interest of the mothers and babies; but are not her political and industrial views purely her own concern? I should be glad to know what readers of THE HOSPITAL think of the above stipulation.—Yours faithfully,

THEODORE HARRIS.

18C Uttoxeter Old Road, Derby.
April 26, 1920.

[The Bermondsey Council is controlled by extremists of the Labour-Socialist type; it has for some time made itself prominent by adding to its proper municipal functions the endeavour to promote political ideas, even to the extent of offering to the Government unasked advice on the conduct of foreign affairs!—Ed. THE HOSPITAL.]

